# Effective diesel removal by a novel electrospun composite nanofibrous membrane with immobilized *Bacillus cereus* LY-1

**DOI:** 10.1039/d2ra06403k

**Published:** 2022-11-29

**Authors:** Yilan Zhang, Xiaoguang Ying, Bo Liu, Bo Yang, Xiao Li

**Affiliations:** College of Chemical Engineering, Fuzhou University Fuzhou Fujian Province 350116 P. R. China lxzwy@fzu.edu.cn

## Abstract

Nanofiber membranes have recently been considered as promising supports for the immobilization of microorganisms due to the simplicity and cost-effectiveness of electrostatic spinning technology and the ability to control fiber morphology, such as obtaining higher surface area and porosity. In this study, electrospun polyvinyl alcohol/sodium alginate/attapulgite (PVA/SA/ATP) nanofiber membrane was prepared as support for immobilized *Bacillus cereus* LY-1 for diesel degradation in an aqueous medium and a significant improvement in diesel removal efficiency was realized. The effect of modified ATP concentration on diesel removal was investigated. The results showed that the nanofiber membranes complexed with cetyl trimethyl ammonium bromide (CTAB) and 1% ATP (w/w) had the best capacity for diesel removing. When the initial diesel concentration was 2 g L^−1^, about 87.8% of diesel was removed by the immobilized LY-1 cells after 72 h. Immobilization of bacteria improves the ability of bacteria to survive in adverse environments. Immobilized LY-1 cells maintain the nature to remove diesel at high salinity or pH range of 6–9. Furthermore, the reusability of the LY-1 cells-immobilized PVA/SA/CTAB–ATP nanofiber membrane was tested. A diesel removal rate of 64.9% could be achieved after 4 times of use. PVA/SA/CTAB–ATP nanofibrous membranes with immobilized LY-1 cells are feasible, economical and environmentally friendly for remediation of diesel contamination in the aqueous medium, and have potential applications in the future.

## Abstract 2

Electrospun PVA/SA/ATP (attapulgite) nanofiber membrane was prepared for *Bacillus cereus* LY-1 immobilizing for diesel degradation in aqueous medium. ATP enhanced diesel removal capacity of bacteria. 87.8% of diesel (2 g L^−1^) was removed after 72 h.

## Graphical abstract

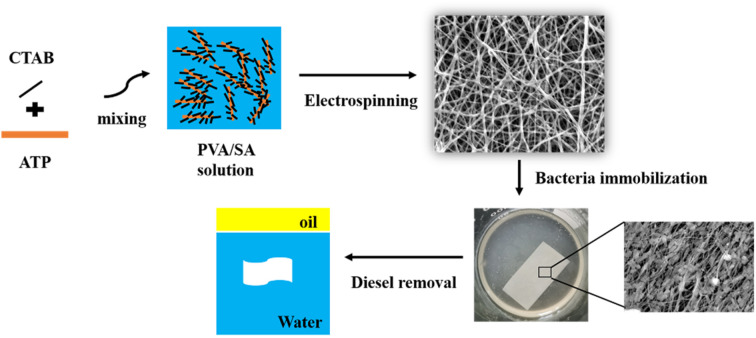

## 1 Introduction

The pollution of water bodies by petroleum hydrocarbons has become a serious environmental problem. Accidental spills of petroleum products often occur during refining, storage and transportation.^1–3^ The International Tanker Owners Pollution Federation Ltd (ITOPF) reported that the total volume of persistent and non-persistent hydrocarbon spills recorded into the environment in 2019 was approximately 1000 tons.^4^ Among hydrocarbons, diesel oil is the most common aquatic pollutant due to oil spills. Diesel oil can be very damaging to both ecosystems and human health.^5^

Currently, the commonly used methods for remediating hydrocarbon-polluted water can be classified as physical, chemical, and biological methods. The physical and chemical methods can rapidly remove the majority of spilled oil. However, they have their own limitations, such as low efficiency, high costs or causing secondary pollution.^6,7^ Bioremediation is an effective, economical and environmentally friendly treatment that involves microorganisms to degrade hydrocarbons. In light of the increasing awareness of environmental protection, biological treatment is increasingly becoming the preferred method for treating petroleum hydrocarbon pollution.^8,9^

Studies carried out on the bioremediation of water were not as many as compared to bioremediation in soils.^4^ The efficient, eco-friendly and cost-effective method for petroleum hydrocarbon removal from aqueous systems remains a challenge. Biodegradation is often limited in unartificial environment by deleterious condition and microorganisms dilution in open water systems. Effective contact between microbial cells and petroleum hydrocarbons floating on the water surface is also a major problem.^10^ The emergence of immobilized cells is a promising approach to solve such problems. Immobilized cells have been studied since 1970s and were developed from immobilized enzyme technology.^11^ Immobilized cell systems are considered to be more advantageous than free cell systems due to the easier separation, possible reutilization of microorganisms, stability against tensions of prohibitive materials and toxic metabolites over long time periods, and preclusion of cell washout at high dilution rates.^3,12–14^

At present, most of the supports for the immobilization of microorganisms used in water remediation applications are made by encapsulation method or porous inorganic materials. However, the preparation process of the embedding method is complicated and costly, with high mass transfer resistance, which affects the growth and metabolism of microorganisms.^15,16^ Support for immobilizing bacteria is expected to provide sufficient space for bacteria to attach and grow and satisfying surface area ratio for cell attachment, mass transfer and degradation.^17,18^ The porosity of the material also plays an important role in biodegradation. High porosity means higher mass transfer efficiency, which also leads to higher diesel removal efficiency. Electrospinning is a special type of fiber manufacturing process. The polymer solution is ejected in a strong electric field, in which electrostatic force is applied to overcome the surface tension of the liquid, producing fibers with diameters in nanoscale.^19,20^ Nanofiber membrane has promising applications in immobilizing bacteria due to their large specific surface area, high porosity, good flexibility, and the ability to control fiber morphology, such as high surface area and porosity.^20,21^ Currently, electrostatically spun nanofibers have been used as supports for other applications, including drug delivery, filtration, enzyme immobilization, and biosensors, among others.^22^ In water treatment, membrane-based support for bacterial degradation has been less studied. Electrospinning membranes with nanostructure for bacteria immobilizing to enhance degradation have not been fully explored.

Many support materials have been used to immobilize microorganisms to improve their biodegradation efficiency. Polyvinyl alcohol (PVA) and sodium alginate (SA) have been widely used in biomedical applications, such as wound dressing, drug delivery and microbial encapsulation, due to their biodegradability, biocompatibility, non-toxicity and low price.^23–27^ PVA nanofiber membrane is difficult to maintain morphology stable in an aqueous solution for a long time. Therefore, it is advisable to cross-link PVA to prevent dissolution when it is used in water treatment. The mechanical strength of the cross-linked membrane increases, but the hydrophilicity is greatly reduced, which is not conducive to the attachment of microbial cells with hydrophilic surfaces.^28,29^ Alginate is a natural anionic linear polysaccharide polymer derived from brown algae.^30^ SA provides PVA films with improved hydrophilicity, swelling ability, elasticity and thermal stability.^23,31^

Composite supports can complement each other in terms of performance and help to improve their applicability.^32^ It has been found that organic support is strengthened and improved in the adsorption of pollutants by adding inorganic substances such as clay, halloysite nanotubes and activated carbon.^18,33–35^ Attapulgite clay (ATP) is an aluminum–magnesium silicate mineral, a rare non-metallic mineral resource. ATP has been widely used in wastewater treatment due to its unique fibrous crystal structure and superior physical and chemical properties such as colloid, adsorption, catalysis and filling.^36,37^

In this study, a novel PVA/SA/ATP nanofiber membrane was prepared by electrostatic spinning technique, through which the petroleum hydrocarbon-degrading strain *Bacillus cereus* LY-1 was immobilized for the removal of diesel fuel from contaminated water. The concentration of ATP and different modified ATP were analyzed according to degrading performance. Moreover, the effects of abiotic factors including pH and salinity on the diesel removal efficiency of immobilized cells were also investigated. In addition, the reusability of bacterial immobilized nanofiber membrane was determined to assess their reuse potential in diesel fuel removal.

## 2 Materials and methods

### 2.1 Materials

PVA (1788, degree of alcoholysis 0.87–0.89) was purchased from Aladdin Biochemicals Technology Co., Ltd. Sodium alginate (SA) was purchased from Sinopharm Chemical Reagent Co., Ltd. Attapulgite (ATP) was supplied by Changzhou Bangding (China). Glutaraldehyde (GA) (25% in H_2_O) and HCl (37%) were purchased from Macklin. Cetyl trimethyl ammonium bromide (CTAB) was purchased from Aladdin Biochemicals Technology Co., Ltd.

### 2.2 Bacterial growth and media

The selected strain *Bacillus cereus* LY-1 was isolated from Indonesian oil sands. It was incubated in Luria–Bertani (LB) medium at 40 °C for 24 h.^38,39^

The minimum salt medium (MSM) contains: KH_2_PO_4_; 2.7 g L^−1^, K_2_HPO_4_; 13.9 g L^−1^, diesel; 2 g L^−1^, NaCl; 1 g L^−1^, yeast extract; 0.5 g L^−1^, NaNO_3_; 1 g L^−1^. This was autoclaved and after cooling 10 mL L^−1^ of each of the following solutions were added: MgSO_4_, 2.5% (w/v); (NH_4_)_2_SO_4_, 10% (w/v); and a trace element solution.^40^

The trace element solution (g L^−1^) contains: ethyl diamine tetra acetate (EDTA); 1, MnSO_4_·H_2_O; 3, FeSO_4_·7H_2_O; 0.1, CaCl_2_·2H_2_O; 0.1, CoCl_2_·2H_2_O; 0.1, ZnSO_4_·7H_2_O; 0.1, CuSO_4_·7H_2_O; 0.01, H_3_BO_4_; 0.01, Na_2_Mo_4_·2H_2_O; 0.01, AlK(SO_4_)_2_; 0.01.^40^

### 2.3 Surface modification of ATP

#### 2.3.1 Acid activation

ATP and 2 mol L^−1^ HCl were added to the beaker at a solid–liquid ratio of 1 : 4, ultrasonicated for 30 min, then magnetically stirred at 40 °C for 5 h, filtered and washed until the pH value of the filtrate remained unchanged. The product was dried at 60 °C for 24 h to get modified ATP (acid–ATP).

#### 2.3.2 Organic modification

Acid–ATP was modified with cetyl trimethyl ammonium bromide (CTAB). Acid–ATP was dispersed in a CTAB solution at a concentration of 6% in an ultrasonic bath for 30 minutes, and then stirred at room temperature for 12 h, filtered and washed until there was no bubble in the filtrate. After that, the powder was washed 3 times with anhydrous ethanol and dried at 60 °C for 24 h to obtain modified ATP (CTAB–ATP).

### 2.4 Preparation of electrospun nano-fibrous membrane

#### 2.4.1 Solution preparation

PVA was dissolved in deionized water with a concentration of 12% by magnetic stirring at 90 °C. A 2% SA solution was prepared by magnetic stirring at room temperature and then mixed with PVA solution at a volume ratio of 7 : 3.^41^ A certain concentration of ATP was added and blended at room temperature by magnetic stirring for 2 h then by ultrasonic dispersion for 30 min and static defoaming to obtain electrospinning solution.

#### 2.4.2 Electrospinning

The solutions were electrospun at a voltage of 18 kV, and the distance between the needle tip to the collector was 13 cm. The rotating drum collector was used to collect the nanofiber at a speed 830 rpm. After the electrospinning process, the prepared nanofibrous membrane was put into a vacuum oven at 60 °C for 12 h to expel any remained solvent.

#### 2.4.3 Cross-linking of nanofibers

GA and 0.1 mol L^−1^ HCl were mixed 1 : 3 by volume as cross-linking agent. Nanofiber membranes were crosslinked at 60 °C for 24 h.

### 2.5 Bacterial cell immobilization

The nanofiber membrane was cut into 2 cm × 4 cm rectangles and placed in 200 mL breakers with 100 mL of LB medium. Then 1 mL of activated bacterial solution was add and the breakers were incubated at 40 °C for 24 h.

In this study, nanofiber membrane with immobilized bacterial was inspected by scanning electron microscopy (SEM) analysis. Freeze-drying was applied for better observation of the immobilized bacteria morphology. Nanofiber membranes were soaked in bacterial cell suspension for 24 h and then transferred to a freeze-dryer (biocool, FD-1C-50) for 24 h to remove water.^42^

### 2.6 Characterization and analytical method

#### 2.6.1 Scanning electron microscopy (SEM)

The morphologies of the prepared nanofibers were investigated by using SEM (Phenom, Pure^+^). Samples were stick to the conductive tapes and coated with a layer of Au before scanning.

#### 2.6.2 FTIR

FTIR spectra of the modified ATP were obtained by ATR-FTIR (Thermo, Fisher, Nicolet iS50, USA).

#### 2.6.3 Characterization of nanofibrous membranes

The membrane sample dimension for the swelling experiment was 2 cm × 2 cm. They were soaked in deionized water for 24 h at room temperature. The swelling ratio of the membrane was determined by measuring the change in weight before and after the swelling. The mechanical properties of the nanofibrous membranes were examined by the texture analyzer (SMS, Ltd Hamilton, MA, USA). Each sample was cut into a dimension of 3 cm × 1.5 cm with a rectangular shape, then tightly fixed with two metal clamps, and measured at the corresponding extension rates (100 mm min^−1^) at room temperature. Experiments were carried out three times, and the results of the experiments were averaged.

### 2.7 Biodegradation experiments

The biodegradation experiments were conducted in the 50 mL MSM medium with diesel as the only carbon source. For free cells, 1% (v/v) of cell suspension (4.88 × 10^8^ CFU per mL) was added to the MSM medium and incubated at 40 °C, shaking at 100 rpm for 72 h. For immobilized cells, 2 cm × 4 cm of nanofibrous membranes containing immobilized bacterial cells were added to the MSM medium and biodegraded under the same conditions. Since diesel oil is volatile, control experiments were carried out using membranes without bacterial cells. All experiments were performed in triplicate. Residual diesel oil was separated from the liquid medium by extraction with petroleum ether (60–90) and determined by GC (gas chromatographic). The diesel oil removal ratio (*R*%) was calculated from eqn (1):1
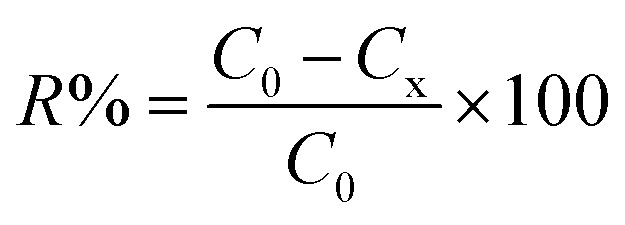
while *C*_0_ (g L^−1^) is the initial concentration before adding bacterial strain and *C*_x_ (g L^−1^) is the residual concentration of diesel oil.

### 2.8 Reusability

Diesel degradation studies were performed four times to assess the reusability of bacterial immobilized nanofiber membrane for an initial concentration of 2 g L^−1^ of diesel oil. Each cycle was performed for 3 days at 40 °C. Before each cycle, the bacterial immobilized nanofiber membranes were washed with sterile distilled water in order to rinse off any unattached or dead bacteria, and then transferred to fresh media containing diesel oil.

## 3 Results and discussion

### 3.1 Morphological characterization of electrospun fibers

**Fig. 1:**
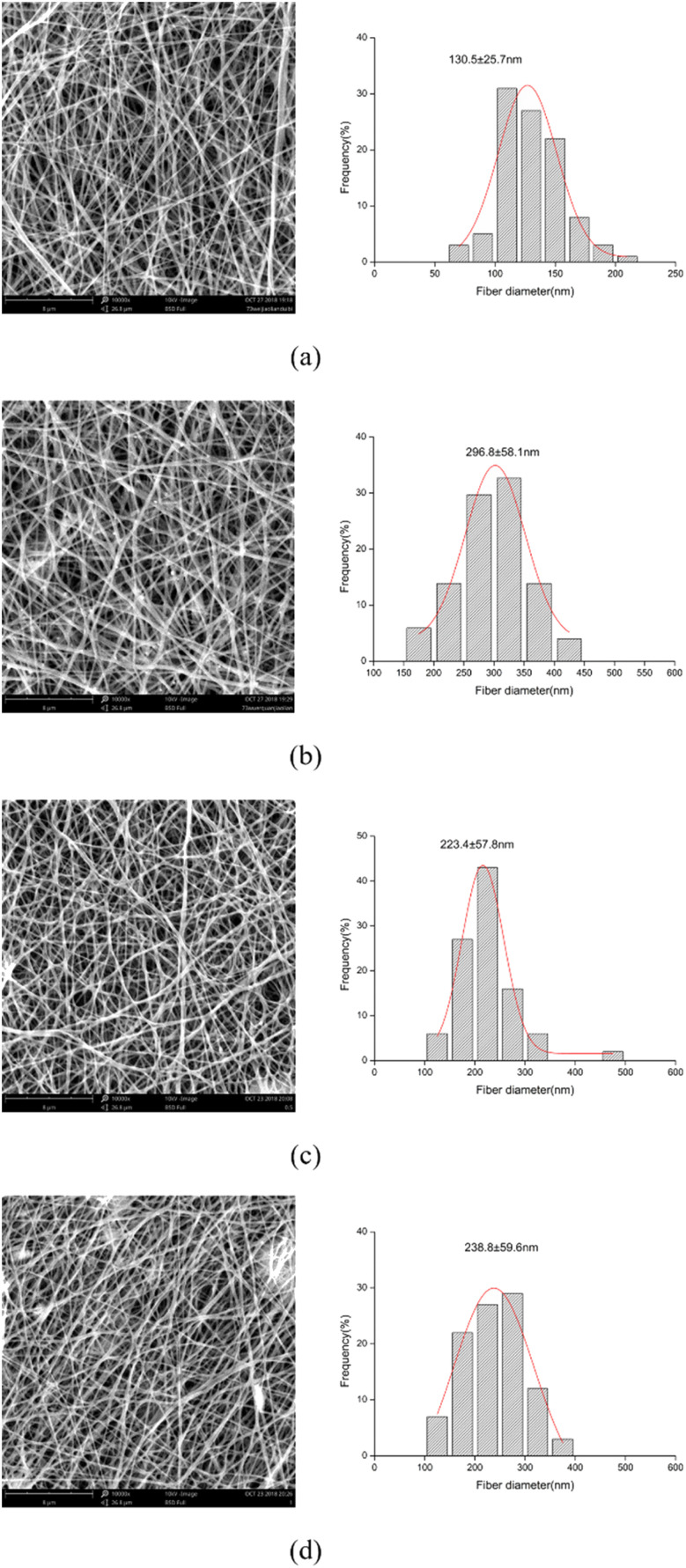
SEM images of electrospun nanofibers and fiber diameter distribution: PVA/SA (a), crosslinked PVA/SA (b), crosslinked PVA/SA/ATP0.5% (c) and crosslinked PVA/SA/ATP1.0% (d).

The morphologies of poly(vinyl alcohol)–sodium alginate with and without attapulgite clay nanofibers were investigated using scanning electron microscope (SEM) as shown in Fig. 1. The degradation experiments of nanofiber membranes with ATP concentrations of 0.5% (PVA/SA/ATP0.5%) and 1% (PVA/SA/ATP1.0%) were investigated. PVA/SA/ATP solution was successfully electrospun into bead-free and uniform fibers with smooth morphology. The addition of ATP affects the size and distribution of the cross-linked nanofiber membrane to different extends. The average diameters of the ATP nanofiber membranes containing 0.5% and 1% were 223.4 nm and 238.8 nm, respectively, indicating a large superficial area of the membrane which enriches adhesion properties.

The cross-linked PVA/SA fibers (Fig. 1(b)) has a very obvious change, with signs of fusion at the overlap of the fibers, which are no longer distinct from each other. Moreover, the diameter of nanofiber membrane increased with increasing the concentration of ATP. The addition of ATP to the PVA/SA solution the viscosity of the solution, resulting nanofibers with larger diameters during electrospinning.^43^ In addition, it can be noticed that the nanofiber membrane shows a web-like structure with good cell attachment potential and is suitable for substrate and nutrient transfer.

### 3.2 FTIR

**Fig. 2:**
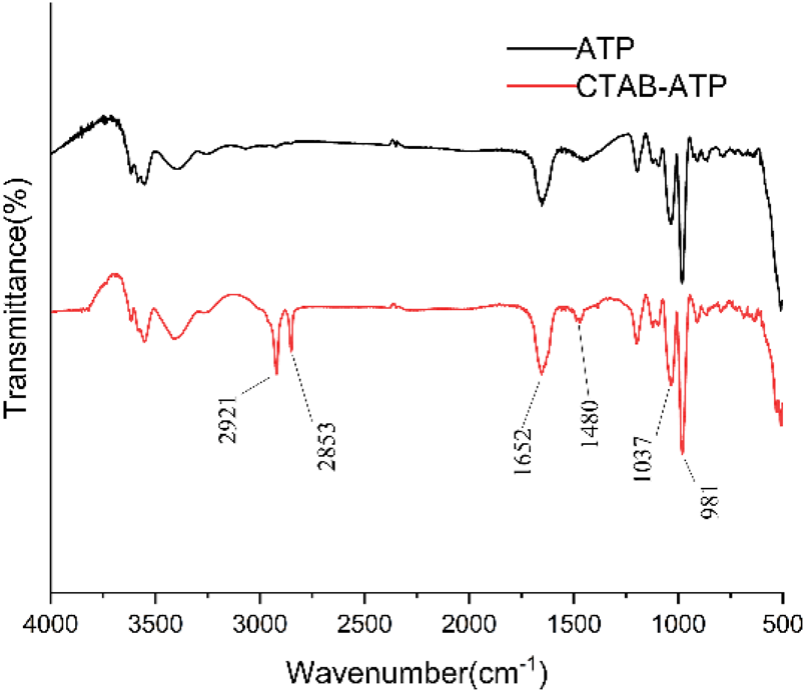
FTIR spectra of unmodified ATP and modified ATP.

The FTIR spectra of untreated ATP and CTAB–ATP after acid activation are shown in Fig. 2. The peaks appearing at 1652, 981 cm^−1^ wave numbers did not disappear, representing that the Si–O–Si and Al–O–Si bonds were not broken and the tetrahedral structure of ATP was intact. It indicates that the acid activation did not lead to the collapse of ATP structure.^44,45^ Symmetric stretching vibration peaks of –CH_3_ and –CH_2_ were observed at 2853 cm^−1^ and 2921 cm^−1^ for CTAB–ATP.^46^ It means that alkyl groups have been incorporated on the surface of the modified ATP.

### 3.3 Swelling ratio and mechanical properties

**Fig. 3:**
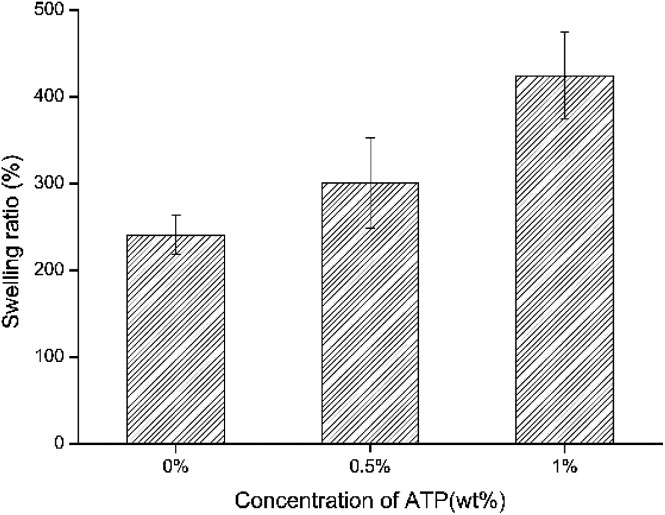
Weight–swelling ratio for different prepared nanofiber membranes.

The results of the swelling rate of different prepared nanofiber membranes in distilled water are shown in Fig. 3. The swelling ratio of PVA/SA/ATP nanofiber membranes increased from 241% to 424% when the concentration of ATP was increased from 0% to 1.0%. This was mainly attributed to that ATP was one of the most typical hydrophilic materials with excellent water absorption and water retention capacity.

**Fig. 4:**
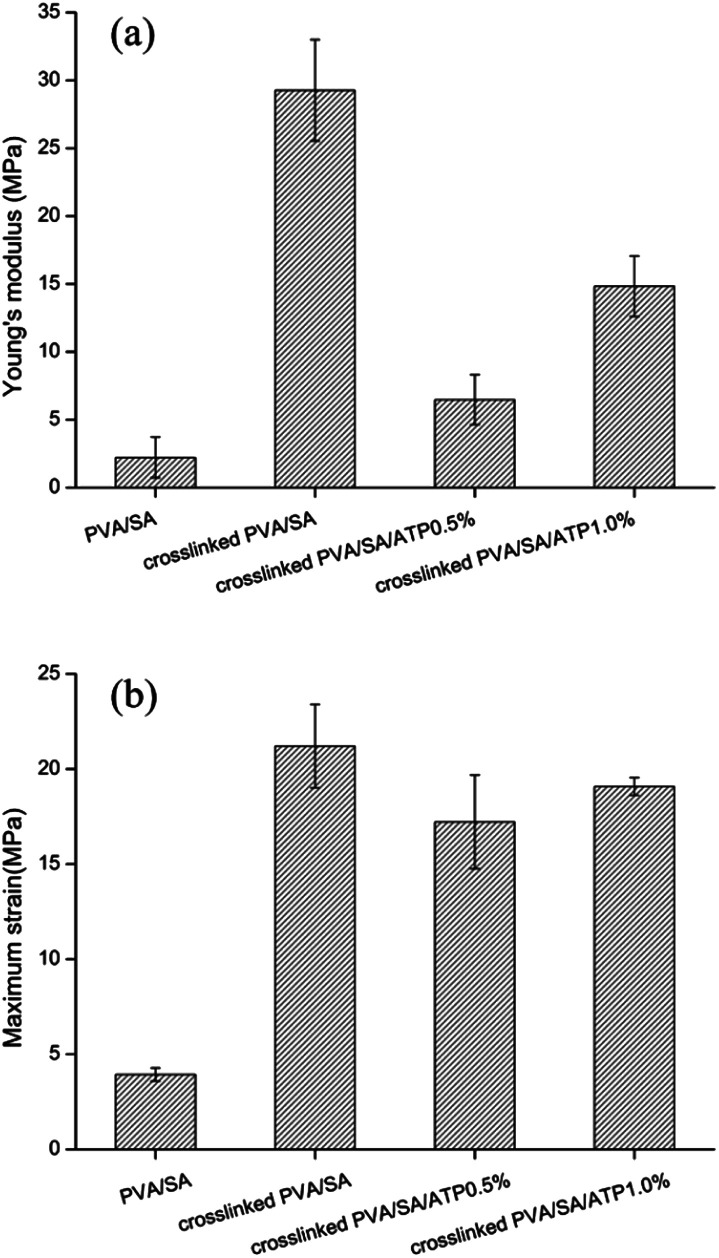
Young's modulus (a), maximum strain (b) of different composition of nanofibers.

The mechanical properties of microbial cell supports are crucial factors for reuse. The Young's modulus and maximum strain (at tensile strength) of the nanofiber membranes are presented in Fig. 4(a and b). The cross-linking of the polymers enhanced the tensile mechanical strength of PVA/SA nanofibers. The maximum strain increased from 3.938 to 21.206 MPa and the Young's modulus increased from 2.215 to 29.267 MPa by the formation of cross-linking points. The addition of ATP caused a significant decrease in Young's modulus of the cross-linked nanofiber membrane. The Young's modulus of PVA/SA/ATP0.5% and PVA/SA/ATP1% were 6.471 and 14.829 MPa, respectively.

### 3.4 Microbial cell attachment

**Fig. 5:**
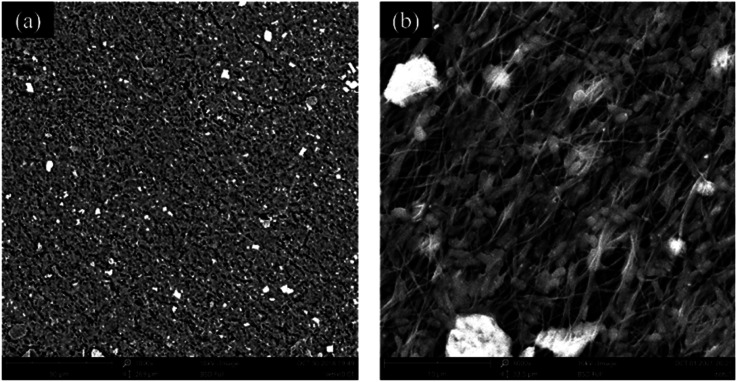
SEM images of PVA/SA/ATP1.0% nanofibrous web after immobilization of *Bacillus cereus* LY-1 at two different magnifications ((a) 1000×, (b) 8000×).

To analyze the growth of bacteria on the nanofiber membrane, the immobilization of bacteria on the PVA/SA/ATP (1.0% (w/w) ATP) nanofiber membrane was analyzed by SEM, as shown in Fig. 5(a and b). *Bacillus cereus* LY-1 is easily distinguished by a unique rod-like shape. After 24 h of incubation, bacteria were attached and biofilm layers were formed on the nanofiber membrane. The results showed that the microbial cells attached densely to the nanofiber membranes and the bacteria were able to grow well on the nanofibers.

### 3.5 Diesel oil biodegradation results

#### 3.5.1 Effect of ATP concentration and modified attapulgite on diesel oil removal

**Fig. 6:**
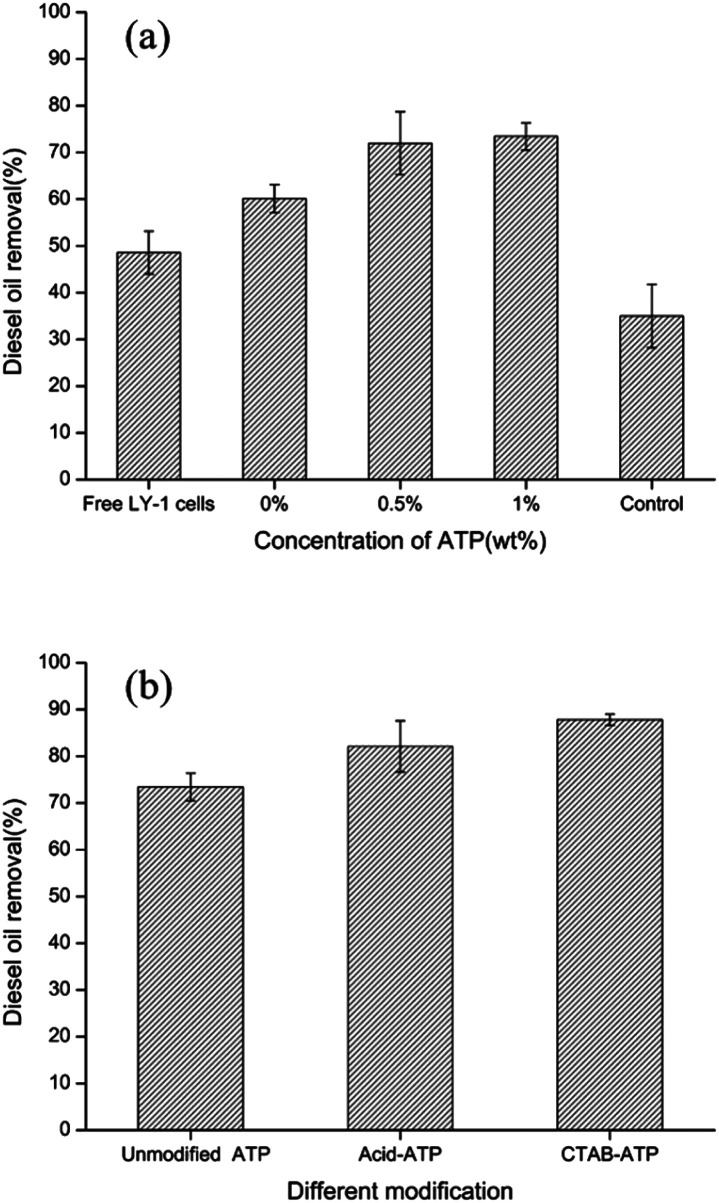
Effects of ATP concentration (a) and modified ATP (b) on diesel oil removal by immobilized LY-1 cells.

To determine the maximum diesel oil removal by the bacteria-immobilized PVA/SA/ATP membrane, the effects of ATP concentrations and modified attapulgite on the biodegradation of diesel oil were investigated. The effect of the ATP concentration on the bacteria-immobilized PVA/SA/ATP membrane on diesel oil removal is illustrated in Fig. 6(a). At the end of the 72 h biodegradation period, the diesel removal rate of the bacteria-immobilized PVA/SA nanofiber membrane was about 60.6%, which was significantly higher than that of free bacteria (48.5%). The maximum diesel oil removal (73.4%) was obtained when the ATP concentration was 1%.

It can be seen in the degradation results that the diesel oil removal rate of immobilized bacteria was significantly higher than that of free bacteria. Immobilized bacteria have strong advantages for applications in aqueous system. Different from the free bacteria, the bacteria-immobilized PVA/SA/ATP nanofiber membrane floated on the surface of water, which allowed bacteria to be in full contact with diesel fuel. The nanofiber membrane holds bacteria together, increasing the density of bacteria on the water surface. Moreover, the diesel degradation rate was further increased by the addition of ATP to the composite nanofiber membrane. The porous structure and large specific surface provide ATP with good adsorption properties. ATP is one of the most typical hydrophilic materials with excellent water absorption and retention capacity.^47–49^ The hydrophilicity of nanofiber membrane is important to immobilize microbial cells with a hydrophilic surface. Since crosslinking apparently reduced the hydrophilicity of PVA. The addition of ATP may be beneficial to bacterial cells growth on the surface of the support.

Fig. 6(b) shows the effect of different modifications of ATP on diesel removal. The diesel removal rate of PVA/SA/acid–ATP nanofiber membrane was 82% after 72 h. The maximum diesel removal rate achieved by PVA/SA/CTAB–ATP nanofiber membrane was 87.8%. Therefore, subsequent experiments were carried out using a CTAB–ATP concentration of 1%.

The results indicated that modified ATP can enhance diesel removal of immobilized bacteria. ATP can be modified in many ways, in this study ATP was modified in two ways: acid modification and organic modification. Natural ATP contains impurities, such as carbonates and metals, which have some mineralogical limitations and weaken the physicochemical properties of minerals.^50^ The acid partially dissolves these impurities, thus increasing the specific surface area, pore size and porosity. Moreover, H^+^ displaces some of the cations in ATP to increase the specific surface area of ATP.^51^ Many researches have modified the surface of clay minerals by introducing long-chain organic surfactants to achieve high adsorption of pollutants.^52^ CTAB, a cationic surfactant, was selected as the modifier of ATP in this study. Modification of ATP with surfactants is mainly due to the ability of surfactants to change particle wettability and reduce water–oil interfacial tension.

#### 3.5.2 Effect of membrane size on diesel oil removal

**Fig. 7:**
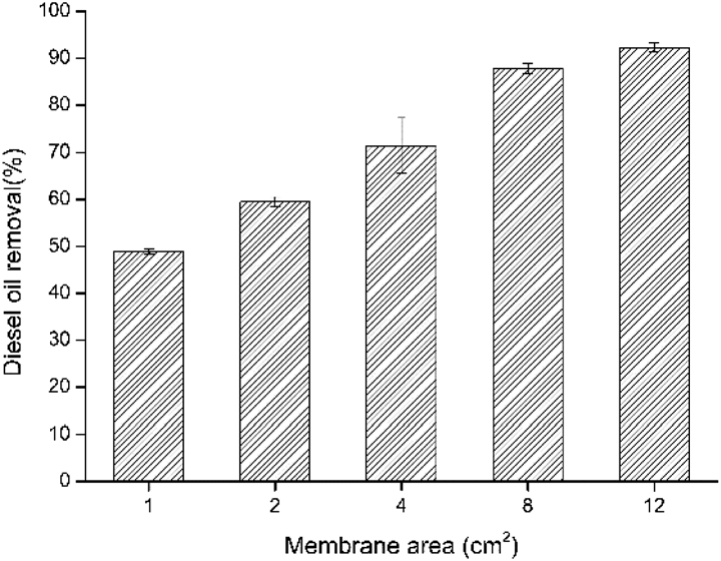
Effect of membrane area on diesel oil removal by immobilized LY-1 cells.

As illustrated in Fig. 7, effect of PVA/SA nanofiber membrane area was investigated using a diesel concentration of 2 g L^−1^ within 3 days degradation time. The results showed an obvious effect of nanofiber membrane area on diesel degradation. The diesel removal rate was increased with increasing the pieces area of the nanofiber membrane as 1 cm^2^, 2 cm^2^, 4 cm^2^, 8 cm^2^ and 12 cm^2^. However, the diesel removal per unit area of nanofiber membrane decreased with an increase in membrane area (Table 1). The special structure of nanofiber membranes provides a large surface area and porosity, creating an advantage for the attachment of bacteria and exposure to contaminants in the aqueous medium. A larger membrane area means that more bacteria adhere to the surface of membrane and contact with the diesel fuel, leading to an increase in diesel oil removal efficiency.

**Table 1:** Diesel removal (mg cm^−2^) by nanofiber membranes of different areas

Membrane area	1 cm^2^	2 cm^2^	4 cm^2^	8 cm^2^	12 cm^2^
The diesel removal (mg cm^−2^)	46	30	21	11.5	5.9

### 3.6 Effect of various degradation conditions

**Fig. 8:**
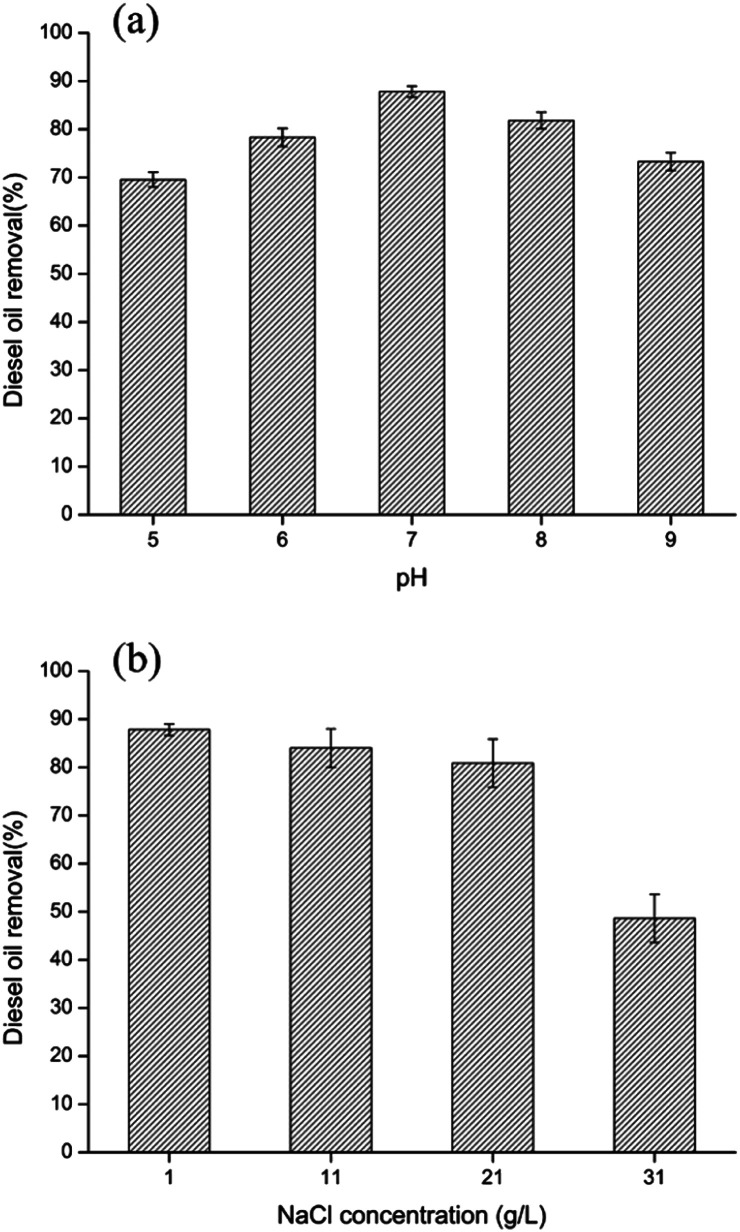
Effect of initial pH (a) and salinity (b) on diesel oil removal by immobilized LY-1 cells.

The growth and degradation ability of bacteria are influenced by various environmental factors. Therefore, the effect of pH value (5, 6, 7, 8, 9) on diesel oil removal using bacteria-immobilized PVA/SA/CTAB–ATP nanofiber membrane was investigated (Fig. 8(a)). At pH values of 5–9, the effect of diesel removal activity of immobilized cells is not significant, with only a slight decrease in diesel removal rate. The diesel removal rate of immobilized bacteria at pH = 5 and 9 were 69.6% and 73.3% respectively, still maintaining high diesel removal rates. In addition, the diesel oil removal under different salinity was investigated (Fig. 8(b)). Additional 1, 2 and 3 wt% NaCl was added to the original MSM medium. The results showed a significant decrease in diesel removal at high salinity of 3 wt%, with a diesel removal rate of 48.6%. Under acidic, alkaline and high salinity conditions, bacterial growth and surfactant secretion was inhibited, leading to the decrease of diesel removal rate. The diesel oil removal activity of immobilized bacteria showed no obvious change at salinities less than 2 wt%, and diesel removal was approximately 80–87% after 72 h. These results indicated that bacteria immobilized in PVA/SA/CTAB–ATP nanofiber membranes were more tolerant to unsuitable environments.

### 3.7 Reusability of immobilized LY-1 cells

**Fig. 9:**
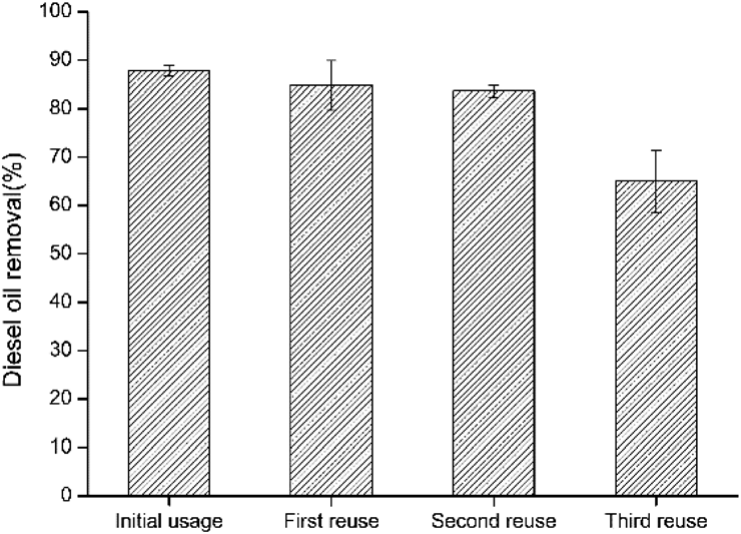
Reusability test results of the four usages on diesel oil removal by immobilized LY-1 cells.

Reusability was studied on the resulted bacteria-immobilized PVA/SA/CTAB–ATP nanofiber membrane for three times after the initial usage. As illustrated in Fig. 9, the diesel removal rate decreased by increasing the reusability usage number. The high diesel removal rate of immobilized bacteria was maintained after the second reuse. After the fourth use (third reuse), the diesel removal rate was approximately 64.9%, indicating that the bacteria-immobilized PVA/SA/CTAB–ATP nanofiber membrane could maintain its biodegradation capacity after several times of usage. The decrease in diesel removal efficiency after repeated use may be due to the detachment of some bacteria from the nanofiber membrane during washing, or the death of some bacteria due to lack of nutrients.

### 3.8 Fiber morphology after reusability test

**Fig. 10:**
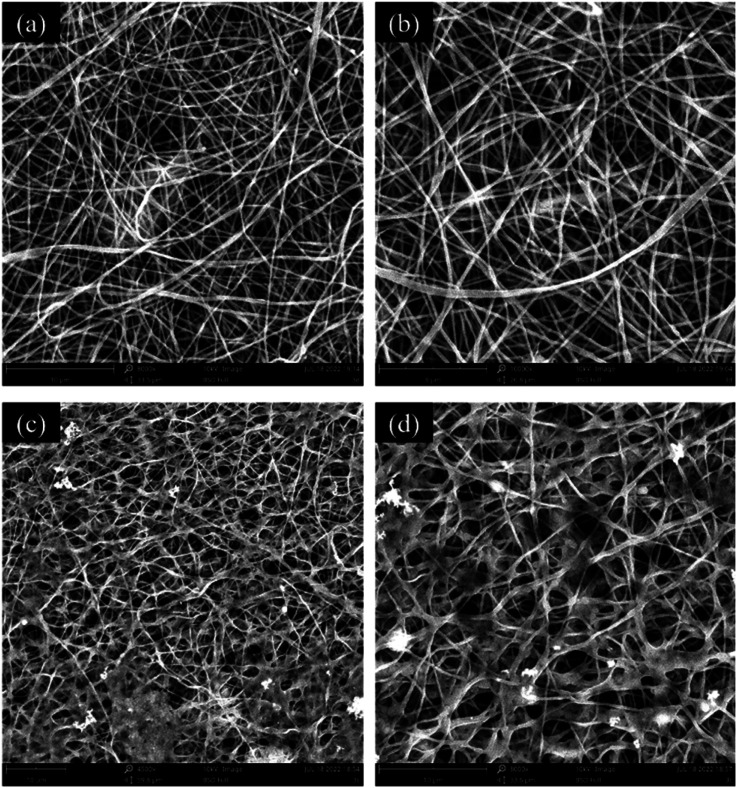
SEM images of PVA/SA/CTAB–ATP nanofibers after reusability tests; reusability test without bacteria (a and b), reusability test with bacteria (c and d).

To observe the changes in the morphology of PVA/SA/CTAB–ATP nanofibers after diesel oil degradation, the membranes were characterized by SEM after the reusability tests. The morphology of the nanofibers without immobilized bacteria is shown in Fig. 10(a and b). The nanofibers swelled due to water absorption. Nevertheless, they still maintained their smooth and uniform morphology with no obvious signs of damage or dissolution observed. The morphology of the bacteria-immobilized nanofiber membrane (Fig. 10(c and d)) confirmed that the fibrous morphology and bacteria were retained.

## 4 Conclusions

In this study, a novel nanofiber membrane was successfully prepared as a bacterial support for the removal of diesel oil from the aqueous medium. The addition of modified ATP to the PVA/SA matrix greatly enhanced the diesel removal capacity of immobilized bacteria. Approximately 87.8% of diesel oil removal could be achieved by the bacteria-immobilized PVA/SA/CTAB–ATP nanofiber membrane after 72 h. Furthermore, the immobilized bacteria showed good diesel removal capacity under unfavorable environmental conditions, including pH and salinity. Moreover, the bio-membrane is reusable and simple to operate. Therefore, the bio-membrane has potential applications in the treatment of diesel-contaminated wastewater.

## Conflicts of interest

The authors declare that they have no conflict of interest.

## Supplementary Material
